# Clinical Predictors and Prediction Models for rFVIII-Fc Half Life in Real-World People with Severe Hemophilia A

**DOI:** 10.3390/jcm12062207

**Published:** 2023-03-13

**Authors:** Chia-Yau Chang, Shyh-Shin Chiou, Te-Fu Weng, Pei-Chin Lin, Shiue-Wei Lai, Chen-Hua Tsai, Yen-Lin Liu, Jung-Tzu Ku, Yu-Mei Liao, Jia-Ruey Tsai, Shu-Hsia Hu, Chao-Neng Cheng, Yeu-Chin Chen

**Affiliations:** 1 Department of Pediatrics, School of Medicine, College of Medicine, Taipei Medical University, Taipei 110, Taiwan; changtiang@yahoo.com.tw (C.-Y.C.);; 2 Division of Pediatric Hematology/Oncology, Department of Pediatrics, Taipei Medical University Hospital, Taipei 110, Taiwan; 3 Hemophilia Center, Taipei Medical University Hospital, Taipei 110, Taiwan; 4 Department of Pediatrics, School of Medicine, College of Medicine, Kaohsiung Medical University, Kaohsiung 807, Taiwan; 5 Division of Hematology and Oncology, Department of Pediatrics, Kaohsiung Medical University Hospital, Kaohsiung 807, Taiwan; 6 Division of Pediatric Hematology/Oncology, Department of Pediatrics, Chun-Shan Medical University Hospital, Taichung 408, Taiwan; 7 Division of Hematology/Oncology, Department of Internal Medicine, Tri-Service General Hospital, National Defense Medical Center, Taipei 114, Taiwan; 8 Hemophilia Care and Research Center, Tri-Service General Hospital, Taipei 114, Taiwan; 9 Department of Hematology/Oncology, Cheng Hsin General Hospital, Taipei 112, Taiwan; 10 Division of Hematology/Oncology, Taipei Medical University Hospital, Taipei 110, Taiwan; 11 Department of Pediatrics, National Cheng Kung University Hospital, Tainan 704, Taiwan

**Keywords:** hemophilia A, half life, pharmacokinetics, predictor, prediction model, rFVIII-Fc, external validation

## Abstract

The half life of recombinant factor VIII-Fc (rFVIII-Fc) for people with hemophilia A (PwHA) varies greatly. Understanding the factors influencing the variation and assessment of rFVIII-Fc half life is important for personalized treatment. Eighty-five severe-type PwHA with rFVIII-Fc treatment receiving an evaluation of half life by the Web-Accessible Population Pharmacokinetic (PK) Service—Hemophilia during 2019–2021 were retrospectively enrolled. The 50-patient PK profiles before 2021 were used for analysis and developing prediction models of half life, and the 35-patient PK profiles in 2021 were used for external validation. The patients in the development cohort were aged 8–64, with a median rFVIII-Fc half life of 20.75 h (range, 8.25–41.5 h). By multivariate linear regression analysis, we found two, four, and five predictors of rFVIII-Fc half life for the blood groups non-O, O patients, and overall patients, respectively, including baseline VWF:Ag, BMI, VWF:activity/VWF:Ag ratio, body weight, O blood group, inhibitor history, HCV infection, and hematocrit. The three prediction equations of rFVIII-Fc half life (T) were respectively developed as T for non-O group patients = −0.81 + 0.63 × (BMI, kg/m^2^) + 6.07 × (baseline VWF:Ag, IU/mL), T for O group patients = −0.68 + 13.30 × (baseline VWF:Ag, IU/mL) + 0.27 × (BW, kg) − 1.17 × (BMI, kg/m^2^) + 16.02 × (VWF:activity/VWF:Ag ratio), and T for overall patients = −1.76 + 7.24 × (baseline VWF:Ag, IU/mL) − 3.84 × (Inhibitor history) + 2.99 × (HCV infection) − 2.83 × (O blood group) + 0.30 × (Hct, %), which explained 51.97%, 75.17%, and 66.38% of the half life variability, respectively. For external validation, there was a significant correlation between the predicted and observed half lives in the validation cohort. The median half life deviation was +1.53 h, +1.28 h, and +1.79 h for the equations of non-O group, O group, and overall group patients, respectively. In total, eight predictors influencing rFVIII-Fc half life were identified. Prediction equations of rFVIII-Fc half life were developed for the non-O and O blood groups and overall PwHA with a good degree of external validation. The equations could be applied to patients aged 8–64 without the need for PK blood sampling and clinically valuable for personalized therapy.

## 1. Introduction

Hemophilia A is the second-most common inherited bleeding disorder and only less frequent than von Willebrand disease (VWD). The common symptoms in people with hemophilia A (PwHA) include joint and muscle bleeding, and repeated joint bleeding could damage joints even in early life. Regular prophylaxis can reduce the joint bleeding rate and promote joint protection, and it has been the standard therapy for severe-type PwHA since 2007 [1,2,3]. However, standard half life (SHL) factor VIII (FVIII) treatment twice or thrice weekly for PwHA on prophylaxis brings about an injection burden and an impact on adherence [4,5]. Extended half-life (EHL) recombinant factor VIII-Fc (rFVIII-Fc) was launched in 2014 and greatly reduced the injection burden and increased prophylaxis rates [6]. Besides markedly decreasing joint bleeding, it has been reported that rFVIII-Fc prophylaxis improves both joint health and function over time in PwHA [7,8]. Many reports revealed that individualized prophylaxis could prevent bleeding more effectively [9,10,11]. Assessing pharmacokinetics (PK), including FVIII half life, for individual PwHA is important for personalized prophylaxis and regimen adjustments [12].

Inter-individual variation in the half life of SHL FVIII was reported to be evident and related to the pre-infused von Willebrand factor (VWF) level [13,14]. Variation in the SHL FVIII half life was also related to the clinical phenotype of severe PwHA on prophylaxis [15,16]. In addition, many authors have mentioned that PwHA with blood group O have shorter infused SHL FVIII half lives than PwHA with blood group non-O [16,17,18,19,20,21,22,23,24]. Moreover, some determining factors related to FVIII half life have been identified, including VWF:Ag and VWF/propeptide ratio [14,23]. Even covariates, such as age and body weight, affecting VWF levels could also affect FVIII half life [14,24,25]. However, genotypes of PwHA or levels of protein C or low-density lipoprotein receptor-related proteins had no influence on the PK of FVIII [23]. EHL FVIII might have similar factors affecting half life. Some authors have reported that the EHL rFVIII half life of patients with blood group O was significantly shorter than that in patients with blood group non-O [24,25]. The A-LONG study identified a correlation between VWF:Ag and rFVIII-Fc half life and found that a higher VWF:Ag level is associated with a longer rFVIII-Fc dosing interval [26]. Carcao et al. reported that the half lives of the EHL products rFVIII-Fc and Rurioctocog alfa pegol are correlated with the VWF:Ag level based on real-world data [27]. However, the question of whether other clinical factors have an impact on rFVIII-Fc half life remains to be investigated.

Although rFVIII-Fc half life can be assessed by the Web-Accessible Population Pharmacokinetic Service—Hemophilia (WAPPS-Hemo), it still requires at least two-point blood sampling, which requires specific sampling intervals, a lead time for reports of FVIII:C levels, and a waiting time for an answer from the WAPPS-Hemo [28,29]. For patients for whom venous access is difficult, the special timing of blood sampling is difficult, or a PK assessment is urgent, a more convenient assessment of rFVIIIFc half life without blood sampling remains an unmet need.

The primary goal of our study was to search for potential predictors of rFVIII-Fc half life from the covariates of real-world clinical practice. The secondary goal was to develop prediction models of rFVIII-Fc half life with external validation that could be clinically more convenient when individualizing regimens for PwHA on rFVIII-Fc.

## 2. Materials and Methods

### 2.1. Patients

rFVIII-Fc was launched in Taiwan in November, 2018. After that, many previously treated PwHA who had been registered at Taipei Medical University Hospital (TMUH) and Tri-Service General Hospital (TSGH)—the two tertiary referral centers for PwHA—switched in succession from SHL rFVIII, including Kogenate-FS, Advate, Xyntha, and Kovaltry, to rFVIII-Fc between 2019 and 2021. Because it was the first time that Taiwanese patients began to use EHL products and because there were no Taiwanese PwHA participating in the clinical trials on rFVIII-Fc, the patients who switched to rFVIII-Fc, at that time, were encouraged to receive a PK assessment by the WAPPS-Hemo. The data on rFVIII-Fc half lives that were extracted from the WAPPS-Hemo were recorded for clinical practice.

The inclusion criteria included people with severe hemophilia A who had received rFVIII-Fc treatment, no matter whether it was on-demand therapy or prophylaxis therapy, and who had received a PK assessment by the WAPPS-Hemo. The exclusion criteria included people with non-severe (moderate and mild) hemophilia A, patients without a PK assessment, patients with current FVIII inhibitors, and patients with a combination of other bleeding disorders, such as VWD. A total of 85 people with severe hemophilia A were eligible and included in the analysis.

The overall cohort of 85 PwHA could be simply divided into two cohorts. Among the 85 patients, there were 50 whose PK data from the WAPPS-hemo were obtained before the end of 2020 and who are referred to as cohort 2019–2020. Regarding the other 35 PwHA who switched to rFVIII-Fc much later, their PK data were obtained in 2021 and we refer to them as cohort 2021. For the purposes of the study, the data on cohort 2019–2020 were used as the development cohort for predictor analysis and the development of prediction models, and the data on cohort 2021 were used as the validation cohort for the external validation of the predictive models. Since hemophilia A is a rare disorder and the number of cases is relatively limited, and since this is a retrospective study, in which it was hard to know ahead of time whether there was an adequate case number, an analysis of the sample size necessary for the development of the study, which is a commonly used method in many prospective studies, was not carried out prior to the study. Therefore, a posteriori analysis would be of interest to assess the power of the results obtained. We used external validation to examine the prediction models developed and assess their applicability by analyzing the median deviation between predicted and observed values. This retrospective observational cohort study was approved by the ethics review board of both institutions (IRB number: A202105193 for TSGH and N202111052 for TMUH).

### 2.2. Patient Characteristics

The charts of all 85 PwHA were reviewed. Clinical data were collected for analysis, including age, body mass index (BMI), body weight (BW), ABO blood groups, hematocrit (Hct), baseline VWF:Ag and VWF:activity, and rFVIII-Fc half lives by the WAPPS-Hemo. VWF:activity means the data obtained via VWF:ACL activity or VWF:RCo. The VWF:activity/VWF:Ag ratio was also used as one of the clinical variables because this ratio can represent the quality or functioning ratio of all the VWF molecules and consequently influence FVIII in PwHA. Inhibitor history was defined as PwHA who had had a transient low-titer inhibitor or high-responder inhibitors eradicated by immune tolerance induction (ITI) therapy before the study period. HCV infection was defined as PwHA with positive anti-HCV antibodies. HIV infection was defined as PwHA with positive anti-HIV antibodies and a PCR for HIV.

### 2.3. Regimen and PK Assessment of rFVIII-Fc

Among the 85 PwHA who had a PK assessment, 83 received their PK assessment during regular prophylaxis (RP) with a regimen of 55–65 iu/kg every 5 days, 45–50 iu/kg every 4 days, 30–35 iu/kg every 3 days, 35–50 iu/kg twice weekly (every 3 days and every 4 days, alternatively), and 65 iu/kg once weekly, which was performed according to the regulations for the payments of the National Health Insurance program. When the PK assessment was performed, all patients were in a stable condition without recent bleeding. They received at least 2-spot blood sampling on the following spots: trough-level and, additionally, any two or three spots of post-infusion (4–5 h, 24 h, 48 h, 72 h, 96 h, or 120 h). However, data on FVIII:C levels < 1% were abandoned in the calculation of half lives. The blood sampling numbers and times for a PK assessment depend on the convenience of the patient and the clinical physician’s decision. Therefore, some patients received 2-spot sampling while the others received 3-spot or 4-spot sampling. All FVIII:C levels were checked by one-stage assay. All rFVIII-Fc half lives were calculated by the WAPPS-Hemo. The remaining 2 patients received on-demand therapy with rFVIII-Fc and they also received PK assessments with 2-spot blood sampling, including trough-level and post-infusion (12 h). All data on baseline VWF levels were obtained in a stable condition during regular comprehensive care or before rFVIII-Fc infusion when receiving a PK assessment. There were 4 patients whose data on VWF levels were unavailable.

### 2.4. Predictor Investigation and Prediction Model Development with Internal Validation

Clinical variables were analyzed in order to search for potential predictors. By stepwise backward linear regression, independent predictors from clinical covariates could be found. By multivariate linear regression analysis, we attempted to develop prediction models; when successful, internal validation with the development cohort was performed by examining the median deviation from the values of the predicted rFVIII-Fc half life minus the observed rFVIII-Fc half life and by correlation analysis between predicted half life and observed half life values.

### 2.5. External Validation of the Prediction Models

The data on the validation cohort in 2021 were used for external validation of the prediction models established by the development cohort. Additionally, the median deviation from the values of the predicted rFVIII-Fc half life minus the observed rFVIII-Fc half life was recorded for analysis. Correlation analysis between predicted rFVIII-Fc half life and observed rFVIII-Fc half life values was also performed to examine the predictive performance.

### 2.6. Statistics

We analyzed patient data on clinical characteristics by an independent *t* test, the Mann–Whitney test, a Chi square test, and Fisher’s exact test. After that, we studied the relationship between half life and baseline VWF:Ag, baseline VWF:activity, BMI, and BW by Pearson’s correlation analysis. Because many of the covariates interacted with each other, in order to investigate the interdependence of these variables and determine which variables were independent factors affecting rFVIII-Fc half life, we used univariate linear regression (LR) analysis and stepwise-backwards multivariate LR analysis to search for independent predictors affecting FVIII PK. For this, we used SAS and R software version 2.12.0 (Free Software Foundation, Inc., Boston, MA, USA). A *p*-value < 0.05 was considered statistically significant.

## 3. Results

### 3.1. Patient Characteristics of the Development Cohort and Validation Cohort

A comparison of the clinical features of the 50 PwHA in the development cohort and the 35 PwHA in the validation cohort is shown in Table 1. Between the two cohorts, there were no significant differences in age, the ratio of patients aged <20, BMI, BW, the ratio of HCV infection, the ratio of HIV infection, the ratio of inhibitor history, Hct, baseline VWF:Ag and VWF:activity, and rFVIII-Fc half life. However, compared with the validation cohort, the development cohort had a significantly higher ratio of O blood group patients (34.3% vs. 42%, *p* < 0.01 **) and a higher VWF:activity/VWF:Ag ratio (0.93 vs. 0.85, *p* < 0.05 *). Regarding rFVIII-Fc half life, we found a large amount of variation in both the development cohort and the validation cohort (ranging from 8.25–41.5 h to 8–39 h, respectively).

### 3.2. Analysis of the rFVIII-Fc Half Lives in the Development Cohort

**Figure 1:**
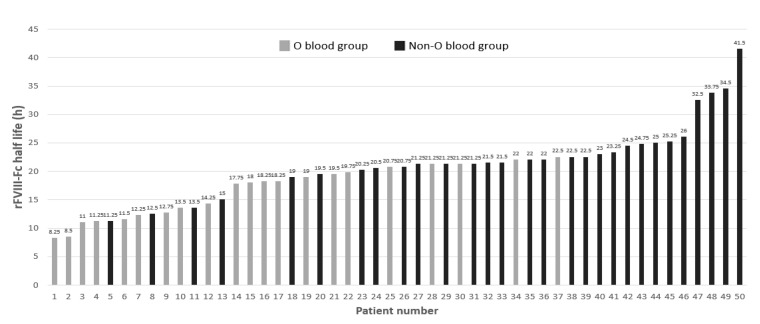
Distribution of rFVIII-Fc half lives marked by the O and non-O blood groups of the development cohort of the 50 people with hemophilia A (PwHA). The numbers above the bars denote the individual rFVIII-Fc half life (h). The gray-colored bars indicate PwHA with blood group O and appear more on the left side of the figure. The black-colored bars represent PwHA with blood group non-O and appear more on the right side.

When we examined the rFVIII-Fc half lives in the development cohort, a large amount of inter-patient variation was found as shown in Figure 1. Interestingly, the top 11 PwHA with the longest half lives (each one ≥ 23 h) were all in the non-O blood group. The twelve PwHA with the shortest half lives (each one < 15 h) were all in the O blood group, except for three patients.

The results of the analysis of patient characteristics and half lives in the development cohort by the Mann–Whitney test are shown in Table 2. Age ≥ 20, BMI ≥ 24 (sorted by mean value), BW ≥ 67.6 kg (sorted by mean value), blood group O, positive HCV infection, baseline VWF:Ag level ≥ 100%, and baseline VWF:activity level ≥ 100% were identified as being significantly associated with a longer half life. The mean half lives of patients with a baseline VWF:Ag level of <100% and ≥100% were 16.58 h and 23.44 h, respectively (*p* < 0.05 *). The mean half lives of patients with a baseline VWF:activity level of <100% and ≥100% were 17.07 h and 24.63 h, respectively (*p* < 0.01 **). However, VWF:activity/VWF:Ag ratio (sorted by mean value), Hct (sorted by mean value), and HIV infection were not significantly associated with half life.

### 3.3. Comparison of the Development Cohort with Blood Group Non-O and Blood Group O

When comparing the characteristics of non-O blood group PwHA and O blood group PwHA, we found no significant difference in age, BMI, BW, Hct, inhibitor history, HIV infection, or HCV infection between the two groups. However, the baseline VWF:Ag level of non-O group and O group patients was 132.1 ± 63.7% and 86.9 ± 19.3%, respectively (*p* < 0.01 **). The rFVIII-Fc half lives of non-O group and O group patients were 22.83 ± 6.46 h and 16.26 ± 4.61 h, respectively (*p* < 0.001 ***).

### 3.4. Correlation Analysis for rFVIII-Fc Half Life

**Figure 2:**
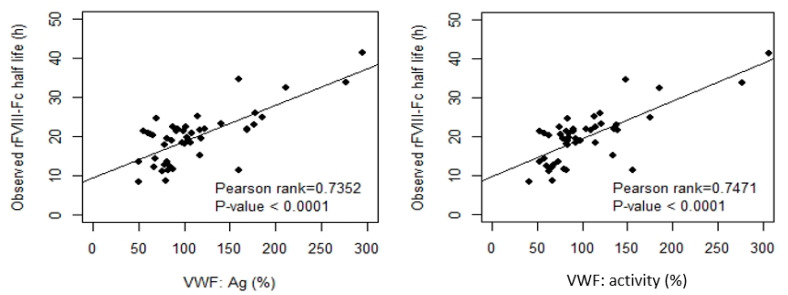
Clinical variables, including body weight (BW), body mass index (BMI), baseline VWF:Ag, and baseline VWF:activity, related to the observed rFVIII-Fc half life in the development cohort (n = 46). Both BW and BMI had a weak or moderate positive correlation to half life (*p*-value < 0.01). Both baseline VWF:Ag and baseline VWF:activity had a strong positive correlation to half life (*p*-value < 0.0001). The drawn lines show the best fit by univariate linear regression.

**Figure 3:**
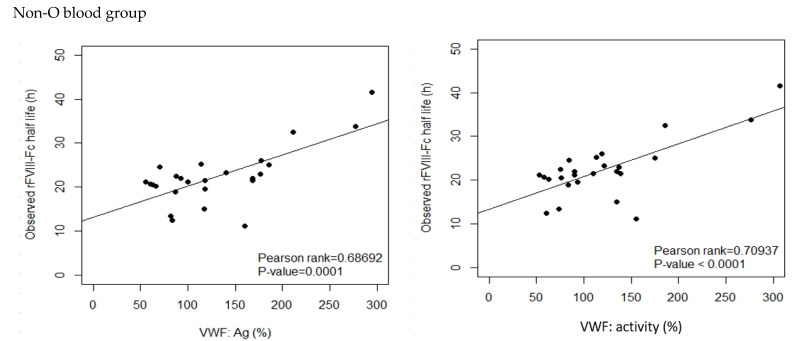

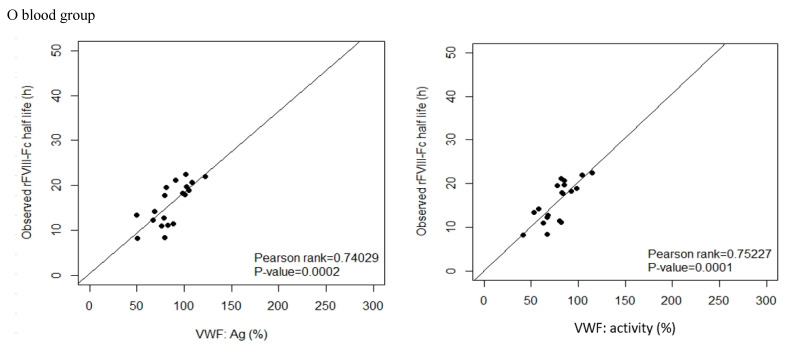
Correlation between the observed rFVIII-Fc half life and baseline VWF:Ag and baseline VWF:activity, respectively, in blood group non-O and blood group O patients of the development cohort. For non-O group PwHA (n = 26), baseline VWF:Ag had a moderate positive correlation to the observed rFVIII-Fc half life (*p*-value < 0.001) and baseline VWF:activity had a strong positive correlation to the observed rFVIII-Fc half life (*p*-value < 0.001). For O group PwHA (n = 20), both baseline VWF:Ag and baseline VWF:activity had a strong positive correlation to the observed rFVIII-Fc half life (*p*-value < 0.001). The drawn lines show the best fit by univariate linear regression.

By Pearson’s correlation analysis of rFVIII-Fc half life in the development cohort, we found that age was a moderate positive correlate to half life (Pearson-rank = 0.6201, *p* < 0.0001 ***). BW and BMI were weak positive correlates to half life (Pearson-rank = 0.4009, *p* = 0.0058 ** and Pearson-rank = 0.3950, *p* = 0.0068 **, respectively), and both baseline VWF:Ag and baseline VWF:activity were strong positive correlates to half life (Pearson-rank = 0.7352, *p* < 0.0001 *** and Pearson-rank = 0.7471, *p* < 0.0001 ***, respectively). The results of the analysis are shown in Figure 2. When the development cohort was separated into an O group and a non-O group for correlation analysis, a similar tendency was found in the two groups but their slopes were different shown as Figure 3. There was a moderate positive correlation of BW and BMI to half life for non-O group patients (Pearson-rank = 0.4552, *p* < 0.05 * and Pearson-rank = 0.5320, *p* < 0.01 **, respectively). BMI was found to have a weak positive correlation to both baseline VWF:Ag and baseline VWF:activity (Pearson-rank = 0.3282, *p* < 0.05 * and Pearson-rank = 0.3427, *p* < 0.05 *, respectively). Given the different slopes in the relationship with rFVIII-Fc half life between blood group O and blood group non-O patients, as well as the higher number of cases with a mix of blood group O and blood group non-O patients, the relationship between the various covariates and rFVIII-Fc half life were analyzed separately for blood group O and blood group non-O and together for the overall development cohort.

### 3.5. Univariate Linear Regression (UVLR) Analysis of Parameters with the Potential for Prediction of rFVIII-Fc Half Life

In our search for valuable parameters to predict rFVIII-Fc half life from the development cohort, we used continuous variables correlated with half life, including age, BMI, BW, baseline VWF:Ag, and baseline VWF:activity, and categorical variables, including ABO blood groups, inhibitor history, HCV infection, and HIV infection. The value of Hct can have an influence on the viscosity of the blood flow and result in a shear force on the VWF, so it was also put into the analysis. The results of the UVLR analysis for the 26 PwHA in the non-O blood group, the 20 PwHA in the O blood group, and all the 46 PwHA are shown in Table 3, Table 4 and Table 5.

### 3.6. Multivariate Linear Regression (MVLR) Analysis for Identifying Predictors and Developing Prediction Models

We further analyzed the data by stepwise-backwards MVLR analysis to assess the interdependence of these parameters. In each step, each parameter with a *p*-value of more than 0.10 was eliminated. The results are shown in Table 6, Table 7 and Table 8.

(1) When analyzing non-O blood group PwHA using this method, age, BW, Hct, inhibitor history, HCV infection, HIV infection, baseline VWF:activity, and the VWF:activity/VWF:Ag ratio were eliminated as predictive parameters. The remaining covariates (baseline VWF:Ag and BMI) were proved to be positive predictors and were used to define Equation (1), which explained 51.97% of all the variability in the rFVIII-Fc half life. As per the mathematical modeling, the rFVIII-Fc half life would increase by 6.07 h whenever the baseline VWF:Ag is increased by 1 IU/mL (100%).
  T (predicted half life of non-O group patients, hrs) = −0.81 + 0.63 × (BMI, kg/m^2^) + 6.07 × (baseline VWF:Ag, IU/mL) (1)
(2) When analyzing O blood group PwHA using this method, age, Hct, inhibitor history, HCV infection, HIV infection, and baseline VWF:activity were eliminated as predictive parameters. The remaining covariates (BMI, BW, baseline VWF:Ag, and the VWF:activity/VWF:Ag ratio) were proved to be predictors and were used to define Equation (2), which explained 75.17% of all the variability in the rFVIII-Fc half life. As per the mathematical modeling, the rFVIII-Fc half life would increase by at least (for it still needs to be added to the impact of the VWF:activity/VWF:Ag ratio) 13.3 h whenever the baseline VWF:Ag is increased by 1 IU/mL (100%).
  T (predicted half life of O group patients, hrs) = −0.68 + 13.30 × (baseline VWF:Ag, IU/mL) + 0.27 × (BW, kg) − 1.17 × (BMI, kg/m^2^) +16.02 × (VWF:activity/VWF:Ag ratio) (2)
(3) When analyzing the overall cohort of PwHA (n = 46) using this method, age, BW, BMI, HIV infection, baseline VWF:activity, and the VWF:activity/VWF:Ag ratio were eliminated as predictive parameters. The remaining covariates (ABO blood group, Hct, baseline VWF:Ag, inhibitor history, and HCV infection) were proved to be predictors and were used to define Equation (3), which explained 66.38% of all the variability in the rFVIII-Fc half-life. As per the mathematical modeling, the rFVIII-Fc half life would increase by 7.24 h whenever the baseline VWF:Ag is increased by 1 IU/mL (100%), decrease by 3.84 h in patients with a positive inhibitor history, increase by 2.99 h in patients with a positive HCV infection test, and decrease by 2.83 h in patients in blood group O.

T (predicted half life of overall patients, hrs) = −1.76 + 7.24 × (baseline VWF:Ag, IU/mL) − 3.84 × (Inhibitor history) + 2.99 × (HCV infection) − 2.83 × (O blood group) + 0.30 × (Hct, %)(3)(For the values of covariates in this equation: 0 if the patient has a negative inhibitor history, 1 if the patient has a positive inhibitor history; 0 if the patient has a negative HCV infection test, 1 if the patient has a positive HCV infection test; and 0 if the patient is in the non-O blood group, 1 if the patient is in the O blood group)

### 3.7. Internal Validation of the Three Prediction Models

**Figure 4:**
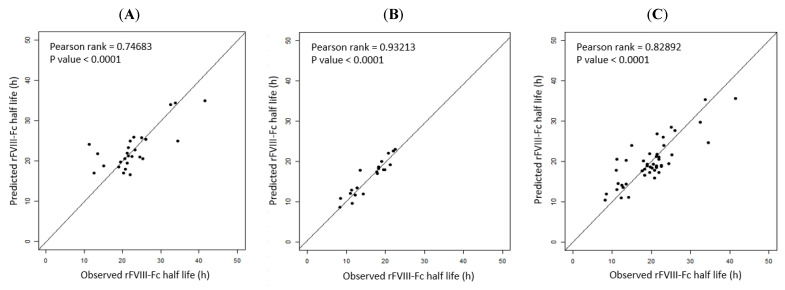
Correlation between the observed and predicted rFVIII-Fc half life values in the development cohort: (**A**) for non-O group PwHA (n = 26) (*p*-rank = 0.74683, *p*-value < 0.0001); (**B**) for O group PwHA (n = 20) (P-rank = 0.93213, *p*-value < 0.0001); (**C**) for the entire cohort of PwHA (n = 46) (P-rank = 0.82892, *p*-value < 0.0001). The drawn lines show the best fit by univariate linear regression.

Regarding the model performance of the prediction equations, differences in the observed and predicted rFVIII-Fc half life values in the prediction models were examined with the data of the development cohort. The median deviation was +0.27 h for Equation (1) of blood group non-O PwHA, −0.04 h for Equation (2) of blood group O PwHA, and +0.79 h for Equation (3) of the overall development cohort, with a standard deviation for the difference of ±4.53 h, ±2.03 h, and ±3.78 h, respectively. Thus, there were a slight tendency to overestimate the rFVIII-Fc half life using Equations (1) and (3) but a slight tendency to underestimate it using Equation (2). When comparing the predicted and observed half lives, more than 80% of the predicted half lives for blood group non-O patients, blood group O patients, and the overall cohort were within a 4.6 h, 2.24 h, and 4.2 h difference of the observed half life, respectively. The predicted half life was within a 4 h difference of the observed half life in 19 (73.1%) of the 26 blood group non-O PwHA by Equation (1), in 19 (95%) of the 20 blood group-O PwHA by Equation (2), and in 33 (71.7%) of the 46 PwHA by Equation (3). The correlation between the predicted and observed rFVIII-Fc half life values is shown in Figure 4A–C.

### 3.8. External Validation of the Three Prediction Models

**Figure 5:**
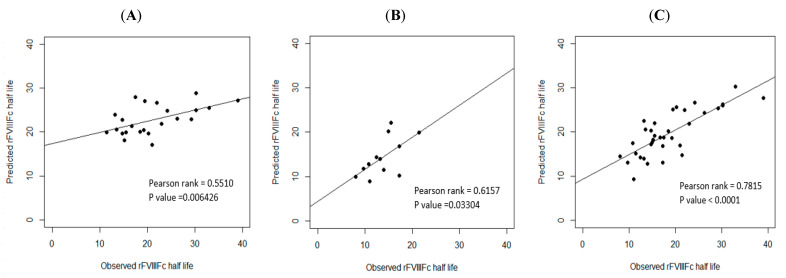
External validation. Correlation between the observed and predicted rFVIII-Fc half life values of the validation cohort: (**A**) for non-O group PwHA (n = 23) (P-rank = 0.5510, *p*-value < 0.01); (**B**) for O group PwHA (n = 12) (P-rank = 0.6157, *p*-value < 0.05); (**C**) for the entire cohort of PwHA (n = 35) (P-rank = 0.7815, *p*-value < 0.0001). The drawn lines show the best fit by univariate linear regression.

In order to examine the predictive performance of the three models, the data on the validation cohort were used for external validation. As calculated by the three prediction equations, the median half life deviation was +1.53 h for Equation (1) of blood group non-O PwHA, +1.28 h for Equation (2) of blood group O PwHA, and +1.79 h for Equation (3) of the overall PwHA cohort, with a standard deviation for the difference of ±6.04 h, ±3.63 h, and ±4.48 h, respectively. Thus, there was a slight tendency to overestimate the rFVIII-Fc half life using Equations (1)–(3). The correlation between the predicted and observed rFVIII-Fc half life values is shown in Figure 5A–C.

## 4. Discussion

In the analysis of our cohort, the real-world data on the rFVIII-Fc half life show a large amount of inter-patient variation (range, 8.25 h–41.5 h) compared with the relatively homogeneous data on the rFVIII-Fc half life in the A-long study (range, 17.4 h–22 h) [26]. Understanding the variation in the PK between clinical trials and the real world is clinically valuable; therefore, we analyzed the clinical factors influencing the variability in rFVIII-Fc half lives. Real-world PwHA may have chronic synovitis, a history of inhibitor, an HCV infection, an HIV infection, pseudotumors, severe obesity, or profound arthropathy. All of these co-morbidities could affect the PK of FVIII and the consequent regimen and effectiveness. In recent years, the level of physical activity with the possibility of subclinical bleeding, as proved by sonography or MRI of joints, in PwHA who reported no bleeding has also been thought to be a determining factor in the pharmacokinetics of FVIII [30,31,32]. Searching for predictors of rFVIII-Fc half life may help us to understand which determinants can impact the half life and help physicians make better decisions on both the initiation of regimens and the adjustment of regimens. The predictors found in our study provide clinical physicians with an insight into the clinical factors impacting rFVIII-Fc half life. Moreover, prediction models of rFVIII-Fc half life have the potential to make predictions that can uncover new mechanisms underlying how rFVIII-Fc half lives are influenced in real-world settings [33]. We developed and externally validated three prediction models to assess the rFVIII-Fc half life of PwHA. The models incorporate common clinical characteristics of PwHA, including BW, BMI, the ABO blood group, and inhibitor history, and routine laboratory data, including Hct, HCV and HIV virus status, baseline VWF:Ag, and baseline VWF:activity. The prediction models developed in our study can use these clinical variables and mathematical models, without the need for blood sampling of the FVIII:C level, to calculate and predict the rFVIII-Fc half life. Despite the convenience of the WAPPS-hemo, with even two-spot blood sampling for the PK of rFVIII-Fc in recent years, there remain some PwHA who have difficulty when undergoing a PK assessment, including pediatric patients, patients for whom venous access is difficult, patients unwilling to undergo blood sampling, patients unable to undergo blood sampling at a specific time point, patients with mental stress regarding blood sampling, and emergency patients who cannot wait for a report on the half life. In addition, the prediction models can be used for the re-evaluation of the PK of PwHA who have experienced changes in VWF levels, BMI values, or Hct values, which could occur due to age or some special condition. Therefore, the prediction models developed in our study may be clinically valuable and applicable to personalized therapy in hemophilia patients.

In our study, those whose age was <20 and those whose age was ≥20 had different mean half lives (13.68 h vs. 21.67 h, respectively, *p* < 0.001 ***). Moreover, in the univariate LR analysis, age was also significantly associated with rFVIII-Fc half life in blood group non-O patients, blood group O patients, and the overall development cohort. These findings are compatible with previous reports that the half life of FVIII and rFVIII-Fc in children with hemophilia A is shorter than that in adult patients [24,27,28,34,35,36]. However, in the MVLR analysis, age was completely eliminated in all three prediction models, indicating that age is not an independent influencing factor for rFVIII-Fc half life. Interestingly, in our linear regression analysis, when we did not put in any data on VWF as a covariate, age became an independent factor for rFVIII-Fc half life in blood group non-O patients, blood group O patients, and the overall development cohort. This phenomenon is very similar to the result of the study by Versloot O. et al., in which age was also found to be an independent factor impacting EHL rFVIII in MVLR analysis when no data on VWF levels as covariates were added [24]. It is well-known that, as one’s age increases, the endogenous VWF level increases [37] and can consequently affect the FVIII half life. Therefore, although age could have an impact on the rFVIII-Fc half life, it should be regarded as a cofounding factor rather than an independent factor.

In our study, baseline VWF:Ag was revealed to be the strongest predictor of rFVIII-Fc half life in all three prediction models, with a *p*-value of 0.001 or <0.001. This result is compatible with previous reports [14,18,26,27,28,34]. It is well known that VWF binding to FVIII serves to stabilize the FVIII molecule and prevent degradation. As most of the FVIII in circulation is in a complex with VWF, the FVIII half life is dependent on that of VWF [26,38]. High baseline VWF:Ag values have been shown to be significantly correlated with increased half life and decreased clearance of FVIII in severe-type PwHA [27,36] In our study, compared with PwHA in the O blood group, those in the non-O blood group had a significantly higher mean baseline VWF:Ag, which is compatible with the previous reports [34,35,36,37,38,39,40], and had a longer rFVIII-Fc half life [34,36].

Interestingly, the correlation analysis showed that there was a strong correlation between baseline VWF:Ag and rFVIII-Fc half life in both blood group O and blood group non-O patients, but the slopes of the two groups of PwHA were different, which might represent different average clearance rates of rFVIII-Fc due to the significantly different average baseline VWF:Ag level [35]. This phenomenon is compatible with previous reports [13,37]; in the report of Fischer K. et al., non-O and O group patients had distinct correlation ranks between VWF:Ag and FVIII half life [14].

Therefore, given the differences between PwHA in the O and non-O groups in terms of baseline VWF:Ag and FVIII half life, the association between various covariates was analyzed separately in linear regression analysis. Our results seem to show that, on the one hand, due to the significantly lower average VWF:Ag in O group patients, other covariates could have an influence on the half life, for which they competed with each other, and, by MVLR analysis, several variables were proved to be predictors, including baseline VWF:Ag, the VWF:activity/VWF:Ag ratio, BW, and BMI, which explained up to 75.17% of the variability. On the other hand, due to the significantly higher VWF:Ag in non-O group patients compared with O group patients, the strong influence of the higher VWF:Ag might overcome the influence of the other potential covariates on the half life seen in the univariate LR analysis. Finally, by MVLR analysis, only baseline VWF:Ag and BMI were proved to be predictors, explaining 51.97% of the variability.

Our study, to the best of our knowledge, is the first time that the VWF:activty/VWF:Ag ratio has been used as a covariate in a linear regression analysis of the rFVIII-Fc half life in hemophilia patients. By MVLR analysis, we found it to be a predictor of the rFVIII-Fc half life in O group PwHA alone and not of that in non-O group PwHA. The VWF:activity/VWF:Ag ratio is usually used to differentiate type 1 and type 2 VWD [41]. This ratio can be seen as being related to the quality of VWF:Ag. None of the members of our PwHA cohort had concurrent VWD. One of the possible explanations for the VWF:activity/VWF:Ag ratio being a predictor of the half life in O group patients but not in non-O group patients is that the markedly higher level of VWF:Ag in the non-O group PwHA made the influence of the VWF:activity/VWF:Ag ratio not as important as it should have been, but that the lower level of VWF:Ag in O group PwHA caused the ratio to be one of the important predictors.

Many authors have found that the O blood group can impact the FVIII half life dependently due to O blood group patients having a lower VWF level and a lower FVIII level [13,14,15,16,17,18,19,20]. Therefore, the O blood group has often been seen as a confounding factor in the variation of the half life. In our cohort, the difference in the mean rFVIII-Fc half life between non-O group patients and O group patients was 6.5 h (22.8 h vs. 16.3 h, *p* < 0.001 ***). This is much longer than the difference in the SHL rFVIII half life in the report of Fischer [15] (nearly 3 h) and in the meta-analytical report of Franchini [17] (2.6 h). However, it is close to the difference in the EHL rFVIII half life of 4 h in the report of Versloot, in which half lives of outliers had been removed [24]. In our study, the difference in the mean baseline VWF:Ag between the non-O and O groups was around 45% (132.1% for the non-O group vs. 86.93% for the O group, *p* < 0.01 **), which is similar to previous reports in which VWF levels were reported to be 25% to nearly 50% higher in healthy persons in the non-O blood group [40,42]. The lower baseline VWF:Ag in our patients in blood group O could explain and contribute to the shorter rFVIII-Fc half life compared with that in the non-O blood group. However, in our study, after the stepwise-backward MVLR analysis removed the influence of confounding factors on the overall cohort (n = 46), the covariate O blood group was revealed to be an inverse predictor despite the fact that the covariate baseline VWF:Ag was also a strong positive predictor. The covariate O blood group per se can have an impact upon the rFVIII-Fc half life and reduce it by 2.83 h independently of baseline VWF:Ag. This means that O blood group can influence the rFVIII-Fc half life independently through an otherwise lower VWF level. Therefore, there may still be other VWF-independent mechanisms through which O blood group can have an impact upon the rFVIII-Fc half life. This result is similar to the finding in Kepa’s report that O blood group and VWF level are simultaneous predictors of SHL FVIII half life and independent of each other in multiple regression analysis [23]. Dino Mehic also reported that blood group O is a risk factor, independent of VWF and FVIII levels, of increased bleeding and bleeding severity in patients with bleeding of an unknown cause [42].

To develop the prediction models, we used stepwise-backwards MVLR analysis, which has been shown to be a better approach than stepwise-forward MVLR analysis by some authors [43,44]. Because the stepwise-forward method does not provide a simultaneous assessment of the effects of all candidate predictors, potential predictors may not be kept in the models [43]. However, a potential limitation of such a model selection strategy is that it could result in overfitting of the model, which means that the model will be too specific to the development cohort and may not be generalizable to the validation cohort or a cohort outside the development cohort [44].

Before prediction models are adopted in clinical practice, both internal validation and external validation are necessary [45]. We used the first cohort (2019–2020) to establish prediction models and used the second cohort (2021) to determine the performance of the models and verify them by analyzing the median deviation for the difference between the predicted and observed half lives and their correlation. The internal validation revealed a strong correlation in the three prediction models. The external validation revealed that there was a strong correlation for the prediction model of the overall cohort (n = 35, P-rank = 0.7815) but a moderate correlation for the prediction models of patients in group non-O and group O (P-rank = 0.5510 and P-rank = 0.6157, respectively). One of the important reasons for the moderate, but not strong, correlations could be the limited number of cases used for the validation (n = 23 for group non-O patients and n = 12 for group O patients). Grant et al. reported that, for datasets of a small to moderate size, when the sample size used for the model’s development is small, the chances of overfitting increase [42]. During the process of external validation, we found that as the number of cases for external validation increased, the *p*-value became more significant and the P-rank of the correlation became stronger. Because hemophilia A is a rare disease with a prevalence of 1/10,000 and the total number of cases in our cohort was relatively small, the case number that could be used for external validation (35 patients in the validation cohort) was quite limited, especially for the non-O and O blood group patients whose case numbers were even smaller (23 and 12, respectively). Therefore, despite the good R^2^ values of the predictive models for non-O and O blood group patients (51.97% and 75.17%, respectively), the P-rank of the correlation between the observed and predicted half lives indicated a moderate, not strong, correlation, which may be due to overfitting. However, despite the small number of cases for external validation, the trend of the results showed that the three prediction models can be applied in and are feasible for clinical practice.

Regarding the clinical applications of our results, any severe-type PwHA on rFVIII-Fc treatment could have their half life calculated through two of the three prediction equations (one by the non-O or O blood group equation and the other by the overall cohort equation). A clinical physician could therefore obtain the two predictive rFVIII-Fc half life values for the prescribed rFVIII-Fc regimen. The R^2^ values of the three predictive models are sufficient for the non-O group (51.97%), O group (75.17%), and overall cohort (65.38%) models. However, it was also found that these predictors did not completely describe the within-group variability. There may be other covariates that have not been put into the linear regression analysis. For example, the level of activity with the possibility of subclinical bleeding that we previously mentioned [30,31] and bone biomarkers [32] may be important predictors of rFVIII-Fc half life that are worthy of further study. That is, the prediction models developed in our study still have room for improvement.

The limitations of our study are as follows. First, our sample size was relatively small for both the development cohort and the validation cohort. This is related to the fact that hemophilia A is a rare disease, rFVIII-Fc is one of several EHL rFVIII products used in hemophilia treatment centers in Taiwan, and other EHL products were not included in the study. Second, all of the observed rFVIII-Fc half life values used in this study were calculated by the population PK model in the WAPPS-hem and not by a classical PK study for FVIII from 11 samples as per the recommendations from the International Society on Thrombosis and Hemostasis [18]. Third, blood sampling for the PK assessment by the WAPPS-Hemo, in our study, was performed in at least two spots. In other words, some PwHA received two-spot sampling, while others received three- or four-spot sampling, which has not been clinically standardized. Fourth, all the PK data were obtained in real-world practice, in which patients received different regimens and dosing frequencies of rFVIII-Fc as per the regulations of the NHI described in the Materials and Methods section. Fifth, in our study, no data on rFVIII-Fc half life in young pediatric patients (aged < 8 years) and in elderly patients (aged ≥ 65 years) were used. Therefore, the application of the three prediction equations was limited to PwHA aged between 8 and 64. Sixth, all the data on baseline VWF levels were retrospectively collected from charts and many of them were collected under a stable condition or during comprehensive care rather than at the point of pre-infusion for FVIII PK. Seventh, we did not have objective data on the levels of physical activity with possible subclinical bleeding in our cohort and, hence, could not put this important variable into the analysis. Finally, we did not put joint scores of PwHA into the analysis, regardless of whether they were Pettersson scores from joint X-ray films, joint image scores from Hemophilic Early Arthropathy Detection with Ultrasound (HEAD-US), or functional scores from the hemophilic joint health score (HJHS). In future studies, joint scores of PwHA, activity levels, and bone biomarkers could be put into the analysis and could provide the predictive models with better R^2^ values.

## 5. Conclusions

In conclusion, we identified eight predictors impacting rFVIII-Fc half life from real-world data, including baseline VWF:Ag, O blood group, inhibitor history, HCV infection, Hct, BMI, VWF:activity/VWF:Ag ratio, and BW. Moreover, we developed three prediction models for O group patients, non-O group patients, and the overall cohort that explained 51~75% of the rFVIII-Fc half life variability and examined their predictive performance through internal and external validation. The prediction models could be clinically applied to PwHA on rFVIII-Fc treatment for the purpose of routine regimen adjustments without blood sampling of FVIII:C levels. We believe that our results are of value to personalized therapy for hemophilia patients and provide insight into predictors of the rFVIII-Fc half life. Regarding the mechanism underlying how these predictors influence the rFVIII-Fc half life, further study is needed in order to clarify the cause–effect relationship and the interactions among these predictors.

## Figures and Tables

**Table 1:** Comparison of the clinical features of the development cohort and the validation cohort.

Clinical Variables Mean ± SD, Range /Case Number (%)	Development Cohort 2019–2020 (N = 50)	Validation Cohort 2021 (N = 35)	*p* Value
Age (year) ^†^ mean ± SD	34.6 ± 15.9	35.5 ± 14.2	0.7999
median, range (y)	33.5, range 8–64	33, range 13–61	
Age § < 20	10 (20)	5 (14.3)	0.4964
≥20	40 (80)	30 (85.7)	
BMI (kg/m^2^) ^†^	24.1 ± 3.4, range 17.1–35	24.4 ± 4.1, range 15.5–33	0.7845
Body weight (kg) ^†^	67.6 ± 14.6, range 31.3–35	70.7 ± 13.2, range 48.3–102	0.3152
ABO blood group ^‡^			
Non-O group	29 (58)	23 (65.7)	0.007 **
O group	21 (42)	12 (34.3)	
Inhibitor history ^‡^			
Negative	43 (86)	32 (91.4)	0.5143
Positive	7 (14)	3 (8.6)	
HCV infection §			
Negative	20 (40)	21 (60)	0.0694
Positive	30 (60)	14 (40)	
HIV infection ^‡^			
Negative	47 (94)	35 (100)	0.2647
Positive	3 (6)	0 (0)	
Hct (%) ^†^	43.8 ± 4.3, range 29.9–52.6	44.7 ± 3.7, range 37.4–52.1	0.3283
Baseline VWF:Ag (%) ^†^	112.5 ± 54.1, range 50–294.7 ^¶^	112.8 ± 43.0, range 48.8–241	0.9750
Baseline VWF:activity (%) ^†^	103.4 ± 52.3, range 41.3–307 ^¶^	97.3 ± 48.7, range 41–243	0.5937
VWF:activity/VWF:Ag ratio ^†^	0.93 ± 0.12, range 0.67–1.21 ^¶^	0.85 ± 0.18, range 0.4–1.25	0.0245 *
rFVIII-Fc half life (h) ^†^	20.1 ± 6.58	18.7 ± 7.1	0.3615
median, range (h)	20.75, range 8.25–41.5	17.3, range 8–39	

^†^, using an independent *t* test; ^‡^, using Fisher’s exact test; §, using a Chi square test; **, *p* < 0.01; *, *p* < 0.05; ^¶^, VWF data from 46 patients (4 had no available data on VWF levels).

**Table 2:** Patient characteristics, clinical variables, and related tests for the rFVIII-Fc half lives in the development cohort of PwHA.

	Case Number (%)	rFVIII-Fc Half Life (h) (Mean ± SD)	*p* Value
Age (year) ^†^	50 (100)		
<20	10 (20)	13.68 ± 4.09	0.0005 ***
≥20	40 (80)	21.67 ± 6.11	
BMI (kg/m^2^) ^†,‡^	50 (100)		
<24	25 (50)	17.52 ± 4.74	0.0078 **
≥24	25 (50)	22.62 ± 7.23	
Body weight (kg) ^†,‡^	50 (100)		
<67.6	28 (56)	18.29 ± 5.82	0.1498
≥67.6	22 (44)	22.34 ± 6.91	
ABO blood group ^†^	50 (100)		
Non-O group	29 (58)	22.83 ± 6.46	0.0003 ***
O group	21 (42)	16.26 ± 4.61	
Hct (%) ^†,‡^	50 (100)		
<43.8	22 (44)	18.42 ± 5.92	0.3251
≥43.8	28 (56)	21.37 ± 6.88	
Inhibitor history ^†^	50 (100)		
Negative	43 (86)	20.80 ± 6.47	0.0878
Positive	7 (14)	15.61 ± 5.78	
HCV infection ^†^	50 (100)		
Negative	20 (40)	16.93 ± 4.88	0.0079 **
Positive	30 (60)	22.17 ± 6.79	
HIV infection ^†^	50 (100)		
Negative	47 (94)	19.90 ± 6.74	0.205
Positive	3 (6)	22.75 ± 1.98	
Baseline VWF:Ag (%) ^†^	46 (100)		
<100 ^‡^	24 (52)	16.58 ± 4.90	0.0108 *
≥100	22 (48)	23.44 ± 6.88	
Baseline VWF: activity (%) ^†^	46 (100)		
<100	29 (63)	17.07 ± 4.59	0.0014 **
≥100	17 (37)	24.63 ± 7.44	
VWF: activity/VWF:Ag ratio ^†,‡^	46 (100)		
<0.93	23 (50)	19.13 ± 6.87	0.7835
≥0.93	23 (50)	20.60 ± 6.82	

^†^, using the Mann–Whitney test; ^‡^, sorted by mean value; ***, *p* < 0.001; **, *p* < 0.01; *, *p* < 0.05; baseline VWF:activity, VWF:ACL activity, or VWF:RCo. The VWF:Ag and VWF:activity ratios are expressed as a % value here, with 100% equal to 1 IU/mL.

**Table 3:** Univariate linear regression (UVLR) analysis of the half lives of non-O group patients, n = 26.

Parameter	Coefficient	95% CI		*p*-Value
Age (year)	0.254	0.110	0.398	0.0013 **
BMI (kg/m^2^)	1.054	0.347	1.761	0.0052 **
Body weight (kg)	0.233	0.041	0.425	0.0194 *
Hct (%)	0.211	−0.520	0.942	0.5562
Inhibitor history ^†^	−4.986	−13.549	3.578	0.2413
HCV infection ^†^	5.481	−0.435	11.397	0.0679
HIV infection ^†^	−0.087	−8.904	8.730	0.9839
Baseline VWF:Ag (IU/mL)	7.351	4.075	10.628	0.0001 ***
Baseline VWF:activity (IU/mL)	5.638	1.848	9.427	0.0052 **
VWF:activity/VWF:Ag ratio	5.036	−16.959	27.031	0.6408

***, *p* < 0.001; **, *p* < 0.01; *, *p* < 0.05; ^†^, 1 if positive, 0 if negative. Baseline VWF:activity denotes VWF:ACL activity or VWF:RCo. VWF:Ag and VWF:activity are expressed as IU/mL here, with 1 IU/mL equal to 100%.

**Table 4:** UVLR analysis of the half lives of the O group patients, n = 20.

Parameter	Coefficient	95% CI		*p*-Value
Age (year)	0.172	0.050	0.294	0.0084 **
BMI (kg/m^2^)	−0.120	−0.766	0.526	0.7017
Body weight (kg)	0.065	−0.066	0.195	0.3119
Hct (%)	0.247	−0.272	0.766	0.3307
Inhibitor history ^†^	−3.141	−8.446	2.165	0.2296
HCV infection ^†^	4.375	0.519	8.231	0.0284 *
HIV infection ^†^	0	-	-	-
Baseline VWF:Ag (IU/mL)	17.572	9.67	25.475	0.0002 ***
Baseline VWF:activity (IU/mL)	17.793	10.076	25.51	0.0001 ***
VWF: activity/VWF: Ag ratio	9.295	−12.657	31.248	0.3854

***, *p* < 0.001; **, *p* < 0.01; *, *p* < 0.05; ^†^, 1 if positive, 0 if negative. Baseline VWF:activity denotes VWF:ACL activity or VWF: RCo. VWF:Ag and VWF:activity are expressed as IU/mL here, with 1 IU/mL equal to 100%.

**Table 5:** UVLR analysis of the half lives of the entire development cohort of patients, n = 46.

Parameter	Coefficient	95% CI		*p*-Value
ABO blood group ^†^	−6.814	−10.385	−3.244	0.0004 ***
Age (year)	0.261	0.161	0.362	<0.0001 ***
BMI (kg/m^2^)	0.756	0.222	1.291	0.0066 **
Body weight (kg)	0.181	0.055	0.306	0.0058 **
Hct (%)	0.123	−0.388	0.633	0.6302
Inhibitor history ^‡^	−5.021	−10.510	0.468	0.0720
HCV infection ^‡^	6.316	2.536	10.096	0.0016 **
HIV infection ^‡^	3.087	−5.147	11.321	0.4539
Baseline VWF: Ag (IU/mL)	9.256	6.664	11.849	<0.0001 ***
Baseline VWF: activity (IU/mL)	7.697	4.397	10.997	<0.0001 ***
VWF: activity/VWF: Ag ratio	7.127	−10.402	24.656	0.4170

***, *p* < 0.001; **, *p* < 0.01; ^†^, 0 if non-O blood group, 1 if O blood group; ^‡^, 1 if positive, 0 if negative. Baseline VWF:activity denotes VWF:ACL activity or VWF:RCo. VWF:Ag and VWF:activity are expressed as IU/mL here, with 1 IU/mL equal to 100%.

**Table 6:** Stepwise-backwards multivariate linear regression (MVLR) analysis of the half lives of non-O blood group patients, n = 26.

Parameter	Coefficient	95% CI		*p*-Value
Intercept	−0.812	−15.133	13.508	0.9076
Baseline VWF:Ag (IU/mL)	6.065	2.749	9.380	0.0010 **
BMI (kg/m^2^)	0.629	0.015	1.243	0.0451 *
	Adjusted-R^2^ = 0.5197			

**, *p* < 0.01; *, *p* < 0.05. VWF:Ag is expressed as IU/mL, with 1 IU/mL equal to 100%.

**Table 7:** Stepwise-backwards MVLR analysis of the half lives of O blood group patients, n = 20.

Parameter	Coefficient	95% CI		*p*-Value
Intercept	−0.683	−15.650	14.285	0.9238
Baseline VWF:Ag (IU/mL)	13.301	6.815	19.787	0.0005 ***
Body weight (kg)	0.268	0.118	0.419	0.0018 **
BMI (kg/m^2^)	−1.166	−1.911	−0.421	0.0045 **
VWF:activity/VWF:Ag ratio	16.023	4.605	27.440	0.0091 **
	Adjusted-R^2^ = 0.7517			

***, *p* < 0.001; **, *p* < 0.01. Baseline VWF:activity denotes VWF:ACL activity or VWF:RCo. VWF:Ag is expressed as IU/mL, with 1 IU/mL equal to 100%.

**Table 8:** Stepwise-backwards MVLR analysis of the half lives of the entire development cohort of patients, n = 46.

Parameter	Coefficient	95% CI		*p*-Value
Intercept	−1.760	−15.900	12.381	0.8027
Baseline VWF:Ag (IU/mL)	7.243	4.632	9.855	<0.0001 ***
Inhibitor history ^†^	−3.844	−7.229	−0.459	0.0270 *
HCV infection ^†^	2.985	0.273	5.698	0.0319 *
O blood group ^‡^	−2.826	−5.512	−0.140	0.0397 *
Hct (%)	0.304	0.0003	0.608	0.0498 *
	Adjusted-R^2^ = 0.6538			

***, *p* < 0.001; *, *p* < 0.05; ^†^, 1 if positive, 0 if negative; ^‡^, 0 if non-O blood group, 1 if O blood group. VWF:Ag is expressed as IU/mL, with 1 IU/mL equal to 100%.

## Data Availability

Data are available upon reasonable request to the corresponding authors.
